# How is self-regulated learning documented in e-portfolios of trainees? A content analysis

**DOI:** 10.1186/s12909-020-02114-4

**Published:** 2020-06-26

**Authors:** R. van der Gulden, S. Heeneman, A. W. M. Kramer, R. F. J. M. Laan, N. D. Scherpbier-de Haan, B. P. A. Thoonen

**Affiliations:** 1 Radboud Institute for Health Sciences, Department of Primary and Community Care, Radboud university medical center, Nijmegen, The Netherlands; 2 Department of Pathology, School of Health Professions Education, School of Cardiovascular Research Institute Maastricht, Faculty of Health, Medicine and Life Sciences, Maastricht University, Maastricht, The Netherlands; 3 Department of Public Health and Primary Care, Leiden University Medical Centre, Leiden, The Netherlands; 4 Radboud Institute for Health Sciences, Department of Radboudumc Health Academy, Radboud university medical center, Nijmegen, The Netherlands

**Keywords:** Electronic-portfolio, Self-regulated learning, Content analysis, Reflection, Feedback, Learning goals

## Abstract

**Background:**

It is assumed that portfolios contribute to self-regulated learning (SRL). Presence of these SRL processes within the documentation kept in portfolios is presupposed in common educational practices, such as the assessment of reflective entries. However, questions can be asked considering the presence of SRL within portfolios. The aim of this study was to gain insight into the documentation of SRL processes within the electronic (e)-portfolio content of medical trainees. SRL consists of numerous processes, for this study the focus was on self-assessment via reflection and feedback, goal-setting and planning, and monitoring, as these are the processes that health professions education research mentions to be supported by portfolios.

**Methods:**

A database containing 1022 anonymous e-portfolios from General Practitioner trainees was used to provide descriptive statistics of the various available e-portfolio forms. This was followed by a quantitative content analysis of 90 e-portfolios, for which, a codebook was constructed to rate the documentation of the included SRL processes.

**Results:**

The numbers of forms in the e-portfolios varied to a great extent. Content analysis showed a limited documentation of reflective entries, and available entries mainly described events and experiences without explanations and context. Feedback was generally limited to comments on what went well and lacked specificity, context and suggestions for future action. Learning goals and plans were short of specificity, but did contain challenging topics and different goals were compatible with each other. 75% of the e-portfolios showed (limited) signs of monitoring.

**Conclusions:**

The e-portfolio content showed limited documentation of SRL processes. As documentation of SRL requires time and asks for a high level of introspection and writing skills, one cannot expect documentation of SRL processes to appear in e-portfolio content without efforts.

## Background

It is assumed that portfolios can contribute to self-regulated learning (SRL) [1–5]. SRL refers to ‘*the degree to which students are metacognitively, motivationally, and behavio*[u]*rally active participants in their own learning process’* ([6], p. 167). It can be seen as an umbrella term that covers a multitude of processes that supposedly influence learning, including skills (e.g. time management), affective constructs (e.g. self-efficacy) and metacognitive processes (e.g. reflection) [7]. Portfolios, ‘*the collection of evidence that learning has taken place’* ([8], p.192), are used to support and document some of these SRL processes. The health professions education literature generally reports the following SRL processes to be supported by portfolios: *self-assessment*, which can be informed internally via *reflection* and externally via *feedback*, *goal-setting and planning*, and *monitoring* [9–11].

The supposed value of portfolios for SRL is one factor explaining the widespread implementation of portfolios in medical training institutes [12], as SRL is considered an essential skill for medical students and physicians [13–15]. Working and learning in a clinical workplace can be unpredictable and sometimes chaotic [16], and it is expected that only those who regulate their learning well are able to keep track of individual educational needs in such a hectic environment [14]. SRL proficiency is thus expected to be of eminence during workplace-based education [14]. Medical educators are therefore trying to identify and optimise tools and procedures that foster SRL during this type of education.

While educators see in portfolios the potential to function as such a SRL fostering tool, users are more sceptical about the educational value of portfolios. The results of studies that rely on user experiences and perceptions show that opinions of learners concerning the support of their portfolio for SRL are mixed at best [17–24]. In some studies, users indicated that their learning is supported by the portfolio [19–21]. However, other studies showed that users experience limited value of portfolios for feedback, reflection, and in achieving learning goals [17, 18, 22–24]. Users indicated, for example, that reflecting via the portfolio is too restrictive and face-to-face feedback gets neglected or compromised by the portfolio [22, 23].

The negative appraisals of users might be prompted by unfavourable experiences with portfolios, that can be accounted for by factors other than SRL, such as the burden and time needed to complete a portfolio [12, 22]. Likewise, unclarity about the purpose of the portfolio can add to negative user perceptions [25]. Trainees have stated that they experience the portfolio as a way to provide evidence of learning to faculty instead of a tool for self-development [23]. On the other hand, considering the comprehensiveness of SRL and the complexities associated with supporting and/or scaffolding SRL [26, 27], it is also possible that portfolios do not contribute to the SRL processes for which they are deployed within health profession education. Studies looking into user experiences and perspectives cannot quantify the extent to which SRL processes are taking place, therefore also other data sources and methods are required when aiming to establish if and how portfolios contribute to SRL processes.

An additional data source is the content of portfolios, which could clarify to what extent the different SRL processes are present in the documentation that learners keep in their portfolio. In common educational practice, such as the assessment of reflections and learning goals and the use of portfolio content as starting point for supervision, the presence of SRL processes in portfolio documentation is presupposed. However, to our knowledge previous studies have only analysed the content of specific (assessment) instruments, such as the mini-CEX, for the presence and quality of documented SRL processes [28–30]. These studies show that the presence and quality of feedback, reflection and action plans documented could be improved substantially. Consequently, there is too little empirical evidence to substantiate the assumption that SRL processes are present in the documentation of portfolios.

The aim of the current study is to gain insight into the documentation of different SRL processes within the e-portfolio content of trainees, thereby focusing on those SRL processes that are, within the field of health professions education, expected to be supported by and documented in portfolios. The research question is: To what extent are SRL processes, specifically self-assessment via reflection and feedback, goal-setting and planning, and monitoring, documented in e-portfolio content?

## Methods

### Context

The setting of this research is the Dutch General Practitioner (GP) speciality training, as provided by the eight institutes related to the eight University Medical Centres in the Netherlands. The formal framework and guidelines of the speciality training, such as the assessment protocol, are similar for the eight institutes. During the 3 years of speciality training, GP trainees learn, while working in general practice (during their first- and third year) and adjacent fields such as psychiatry and hospital emergency care (during their second year). This workplace-based learning is guided by experienced doctors (mostly GPs), who work on site with the trainee and function as supervisors. In addition, GP trainees receive education in peer-trainee groups, during a weekly academic day, which is provided by GP teachers and behavioural scientists at the different institutes.

Trainees are obligated to document information concerning assessment and learning in an e-portfolio. Content and structure of the e-portfolio are based on the research-informed NijMaas guidelines [31]. The mission of these guidelines was to propose an e-portfolio that combines programmatic assessment [32] with support for SRL. The e-portfolio contains eleven unique forms (see Table 1). These eleven forms can be added as often as required by trainees. Alongside the pre-structured forms, trainees can add their own documents to a separate folder of the e-portfolio.

**Table 1:** Description of the different pre-structured e-portfolio forms and their – during design - envisioned supportive value for SRL processes (with ✓ meaning supportive and – meaning no additional support)

Form	Description	Envisioned support for SRL
Advice on advancement	The form can be used by teachers or supervisors to give advice on the advancement of the trainee. Mandatory	Self-assessment, via: Reflection - Feedback ✓ Goal setting & planning - Monitoring ✓
Competency Assessment List (Compass) [33]	The Compass asks to rate trainees progress level of the different competences of the CanMEDS. Feedback that explains the ratings should also be provided. Mandatory	Self-assessment, via: Reflection ✓ Feedback ✓ Goal setting & planning - Monitoring ✓
Decision of advancement	The decision as to whether or not the trainee is permitted to advance to the next internship. Mandatory	Self-assessment, via: Reflection - Feedback - Goal setting & planning - Monitoring -
Declaration of competence	On this form, supervisors can declare to what extent the trainee is competent to perform certain tasks independently. Mandatory	Self-assessment, via: Reflection - Feedback ✓ Goal setting & planning - Monitoring ✓
Internship evaluation	This form should be used by supervisors to evaluate trainees at the end of their internship, stating if the trainee performed (in)sufficiently. Mandatory	Self-assessment, via: Reflection- Feedback ✓ Goal setting & planning- Monitoring ✓
Learning goals and plans	On this form, trainees can formulate their learning goals and the approach that they will take to reach their goals. Feedback and comments can be added at any time. 25 goals can be added on one form. Mandatory	Self-assessment, via: Reflection- Feedback ✓ Goal setting & planning✓ Monitoring ✓
MAAS-Global rating list [34]	Trainees receive feedback on the communication skills they showed during a consultation. The first part of the list is about phase-specific skills (e.g., opening the conversation). The second part rates general communication skills. The items are scored on a 7-point Likert scale. Optional	Self-assessment, via: Reflection- Feedback ✓ Goal setting & planning- Monitoring ✓
Mini-CEX [35]	Trainees select an activity or skill that was observed in daily practice, and describe which aspects they would like to receive feedback on. The supervisor gives feedback according to the CanMEDS competencies. Subsequently, trainees are asked to reflect on this feedback. Optional	Self-assessment, via: Reflection✓ Feedback ✓ Goal setting & planning- Monitoring ✓
Registration of shifts	Trainees must register their mandatory out of hours shifts in the e-portfolio. Mandatory	Self-assessment, via: Reflection- Feedback - Goal setting & planning- Monitoring ✓
Report on appraisal interview	After every appraisal interview, trainees write a report about their experience with regard to the interview. Mandatory	Self-assessment, via: Reflection✓ Feedback - Goal setting & planning- Monitoring ✓
Request for feedback	Trainees can formulate a topic on which they would like to receive feedback from their teacher/supervisor or a colleague. The form is, thereafter, sent to this person, so that the feedback can be provided. Subsequently, trainees are asked to reflect on this feedback. This form was introduced in 2016. Optional	Self-assessment, via: Reflection✓ Feedback ✓ Goal setting & planning- Monitoring ✓

Trainees share their e-portfolio with teachers, supervisors and heads of the GP Training Institute. Every 4 months, teachers and supervisors assess competency development (via a Compass-form, see Table 1), and provide their advice on advancement in the e-portfolio. Besides that, the e-portfolio content is used to inform the annual summative progress decision made by the head of the GP Training Institute. For this, the e-portfolios also contain the results of mandatory progress tests and video-assessments of doctor-patient communications.

Trainees have access to technical instructions of the e-portfolio and the (programmatic) assessment procedures of their institute, which are derived from the national assessment protocol.

### Procedure

A mirror version of the actual e-portfolio database was constructed. In this mirror database anonymous e-portfolios of trainees from three institutes were stored, from introduction of the e-portfolio (2013) to the end of March 2018. The folder containing personal documents could not be anonymised, therefore, only the pre-structured forms were transferred to the mirror database.

Records containing data of users other than trainees, such as administrators, were excluded from analyses. The database also consisted of e-portfolios that were not (appropriately) used due to e.g. trainees transferring to another institute or dropping-out. To exclude these e-portfolios, absence of any Compass-form (an obligatory form that needs to be completed three times a year, see Table 1) was used as exclusion criterion. All e-portfolios (*N* = 128) without a Compass-form were excluded. In addition, e-portfolios of trainees that were already in their second- or third year during implementation of the e-portfolio were excluded, as these trainees in many cases kept using their old paper portfolio alongside the e-portfolio (*N* = 112). This resulted in a final dataset consisting of 1022 e-portfolios.

To consider differences in duration of training between trainees, the duration of e-portfolio use was specified for all e-portfolios by calculating the difference between March 2018 and the date the specific e-portfolio was created. This duration was used to assign the e-portfolios into three cohorts, which globally represented the 3 years of speciality training.

### Design

This study was conducted in two phases using quantitative measures. The first phase, was carried out to provide descriptive statistics of the use of pre-structured forms of the 1022 included e-portfolios. During the second phase, a quantitative content analysis was performed to rate the manifestation of the included SRL processes in the content of 90 e-portfolios.

These 90 e-portfolios were selected using stratified random sampling, to ensure that the three institutes and 3 years of speciality training were equally represented in the sample. It was decided within the research team to select ten e-portfolios per group (institute x year) to keep the rating process feasible. As the e-portfolio was implemented later in one institute there were too little portfolios available to rate material from the third year, so the decision was made to rate fifteen e-portfolios for the first 2 years for this institute.

### Analyses

#### Phase one: number of forms

Data were exported from the mirror database into eleven different data files that signified the eleven unique pre-structured forms. Within these data files, each case represented one completed form. In subsequent steps, single cases were aggregated per e-portfolio, to determine the number of completed forms per e-portfolio. Different descriptive statistics were calculated using IBM SPSS Statistics 25.

An exception was made concerning the form: ‘learning goals and plans’. Since trainees differed to a great extent in the number of learning goals that they formulated on one form (ranging from one to twenty-four), it was considered inconsequential to compare numbers of forms. So, number of learning goals were compared instead. A goal was included if any text was provided within the first of five text boxes (‘description of the learning goal’), relevance of content was not considered at this point.

#### Phase two: content analysis

A quantitative content analysis of e-portfolios was performed, such an analysis consists of ratings made by the use of a deductively designed coding scheme which is elaborated in a codebook [36–38]. The aim of our analysis was to identify the presence of effective SRL behaviour within the documentation kept in the e-portfolios, thereby focusing on the SRL processes that, according to health professions education literature, can be supported by portfolio use: self-assessment, goal setting and planning, and monitoring [9–11]. As there was no existing instrument available that was suitable to rate the presence of these SRL processes within written documentation, we used research literature regarding the different processes to develop our codebook (see Appendix A).

For the development of the codebook, we searched the research literature for descriptions of good practice of the included SRL processes, so we could formulate criteria that can be rated for their presence. We decided to focus on two processes that inform self-assessment - reflection and feedback - instead of self-assessment itself, as this process, which is for the most part cognitively performed, is difficult to objectify [39].

For the formulation of criteria concerning reflection we used the framework of Hatton and Smith that distinguishes different types of writing with an increasing level of reflection [40, 41]. With regard to feedback we used the conditions for effective feedback formulated by Gibbs and Simpson [42]. Literature by Zimmerman was used to formulate the criteria on effective goal setting and planning [43].

The operationalisation of monitoring [12], was challenging due to limited information on good monitoring practice [44, 45]. Therefore, it was decided to complement a single rating item (‘Does the e-portfolio show signs of monitoring?’) with memos of the observed monitoring activities. During the rating of monitoring we focused on the coherence between different elements of the portfolio: to what extent did themes and topics considered during feedback and reflections recur in learning goals and plans, and vice versa.

**Fig. 1:**
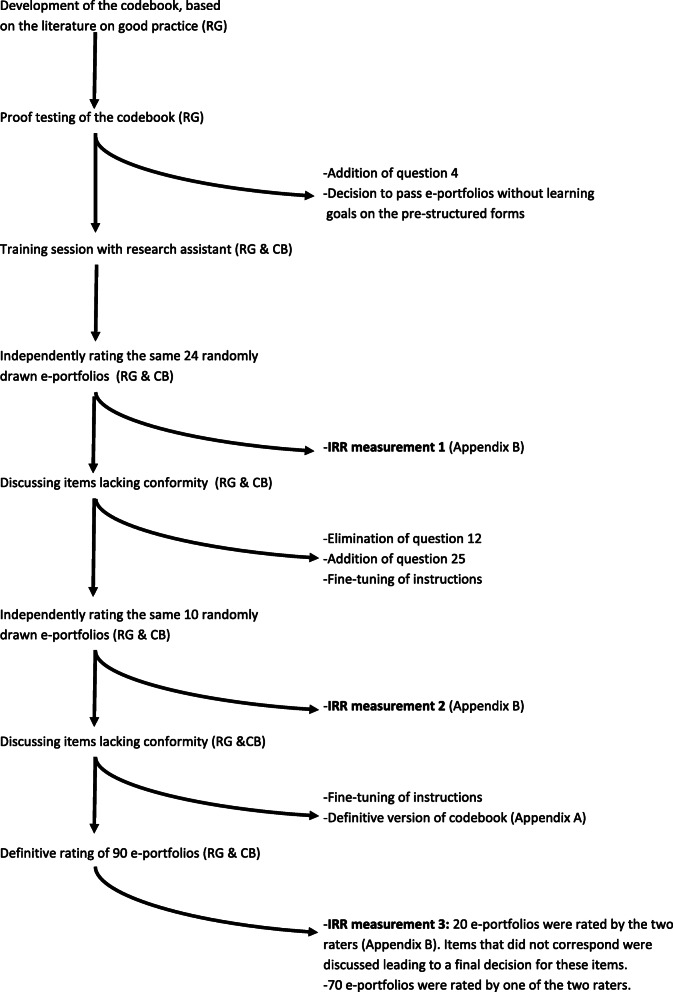
The process that led to the definitive rating of e-portfolios

After a first version of the codebook was approved by all authors, two raters (RG & CB) proof tested the codebook on a sample of e-portfolios, leading to adaptations of the codebook. Subsequently, two sittings were performed during which the raters used the codebook to rate the same randomly drawn e-portfolios, after which adaptations were made. During this process, interrater reliabilities (IRR) were calculated multiple times using Krippendorff’s alpha [46, 47] and percentage of agreement. IRR improved with practice and proved to be acceptable during the definitive rating (see Appendix B). Fig. 1 shows the process that led to the final version of the codebook and the definitive rating of e-portfolios.

During the ratings, the content analysed was confined to material covering one study year, to make sure that the rating period was equal for all e-portfolios included in the content analysis, regardless of the cohort the e-portfolio belonged to. To secure that we rated material that was collected during an entire study year, we chose to include the content from the year prior to the cohort the portfolio belonged to, e.g. content from the first year was included for e-portfolios that belonged to the second year cohort, content from the second year was included for e-portfolios that belonged to the third year cohort, etc.

After the rating was completed the ratings were transferred to an SPSS data file. Frequencies were calculated to gain insight in the number of e-portfolios that met the different SRL criteria for good practice from the codebook.

### Ethical approval and reflexivity statement

The ethical review board of the Dutch Organization of Medical Education (NVMO) approved the study under NERB number 786.

The research team was comprised of a psychologist (RG), physicians (BT, NS, AK, RL) and a health scientist and educationalist (SH). Before starting her psychology study, the first researcher studied mass communication, the field from which content analysis originates. The research assistant (CB), who supported the content analysis, has a background in psychology.

## Results

### Phase one: number of forms

Table 2 provides the descriptive statistics of the number of pre-structured forms used within the e-portfolios. Statistics are split up into the three cohorts, based upon the duration of e-portfolio use. The table shows high standard deviations, representing a considerable difference in numbers of forms present in individual e-portfolios.

**Table 2:** Descriptive statistics of the numbers of pre-structured forms used in the e-portfolios. For each form it is indicated if the form is envisioned to support F(eedback), R(eflection), G(oal-setting) and/or Mo(nitoring). NB: The data comprise the cumulative numbers of forms for the entire period the e-portfolio was in use. Consequently, the statistics of the first cohort (0–12 months) contain data covering up to a maximum of twelve months, whereas the third cohort (> 24 months) covers at least two years of forms

**Form (Mandatory/Optional)**	**Duration of e-portfolio use**	**In how many e-portfolios was the form present (%)**	**Descriptive Statistics:** How many forms were used per e-portfolio	
Advice on advancement (Ma)	≤ 12 months *N* = 119	63.0% (*n* = 75)	μ = 0.91	Sd = 0.86
			Min = 0	Max = 4
	12–24 months *N* = 319	95.9% (*n* = 306)	μ = 4.26	Sd = 2.18
			Mi*n* = 0	Max = 15
*Support for: F, Mo*	> 24 months *N* = 584	81.5% (*n* = 476)	μ =3.59	Sd = 2.46
			Mi*n* = 0	Max = 12
Competency Assessment List (Compass) (Ma)	≤ 12 months *N* = 119	100% (n = 119)	μ = 3.84	Sd = 1.25
			Min = 2	Max = 6
	12–24 months *N* = 319	100% (*n* = 319)	μ = 8.94	Sd =3.47
			Min = 1	Max = 18
*Support for: R, F, Mo*	> 24 months *N* = 584	100% (n = 584)	μ = 18.48	Sd = 5.54
			Mi*n* = 1	Max = 37
Decision of advancement (Ma)	≤ 12 months N = 119	0% (n = 0)	μ = 0	Sd = 0
			Min = 0	Max = 0
	12–24 months N = 319	80.3% (*n* = 256)	μ = 0.91	Sd = .56
			Min = 0	Max = 3
*Support for: -*	> 24 months N = 584	96.1% (*n* = 561)	μ = 1.81	Sd = 0.67
			Min = 0	Max = 4
Declaration of competence (Ma)	≤ 12 months N = 119	0.8% (n = 1)	μ = 0.01	Sd = 0.092
			Min = 0	Max = 1
	12–24 months N = 319	75.2% (*n* = 240)	μ = 0.95	Sd = 0.78
			Min = 0	Max = 5
*Support for: F, Mo*	> 24 months N = 584	76.5% (*n* = 447)	μ = 1.59	Sd = 1.35
			Min = 0	Max = 6
Internship evaluation (Ma)	≤ 12 months N = 119	0% (*n* = 0)	μ = 0	Sd = 0
			Min = 0	Max = 0
	12–24 months N = 319	27.0% (*n* = 86)	μ = 0.37	Sd = 0.66
			Min = 0	Max = 3
*Support for: F, Mo*	> 24 months N = 584	43.3% (*n* = 253)	μ = 0.88	Sd = 1.14
			Min = 0	Max = 4
MAAS-Global rating list (O)	≤ 12 months N = 119	44.5% (*n* = 53)	μ = 2.40	Sd = 3.09
			Mi*n* = 0	Max = 8
	12–24 months N = 319	28.8% (*n* = 92)	μ = 1.61	Sd = 2.90
			Min = 0	Max = 10
*Support for: F, Mo*	> 24 months N = 584	16.8% (*n* = 98)	μ = 0.90	Sd = 2.22
			Min = 0	Max = 15
Mini-CEX (O)	≤ 12 months N = 119	89.1% (*n* = 106)	μ = 10.08	Sd = 10.37
			Min = 0	Max = 44
*Support for: R, F, Mo*	12–24 months N = 319	79.9% (*n* = 255)	μ = 13.86	Sd = 17.75
			Min = 0	Max = 86
	> 24 months N = 584	88.0% (*n* = 514)	μ = 28.91	Sd = 26.84
			Min = 0	Max = 171
Registration of shifts (Ma)	≤ 12 months N = 119	97.5% (*n* = 116)	μ = 8.62	Sd = 2.83
			Min = 0	Max = 14
	12–24 months N = 319	95.3% (*n* = 304)	μ = 17.78	Sd = 5.57
			Min = 0	Max = 31
*Support for: Mo*	> 24 months N = 584	98.3% (*n* = 574)	μ = 29.99	Sd = 10.67
			Min = 0	Max = 49
Report on appraisal interview (Ma)	≤ 12 months N = 119	52.9% (*n* = 63)	μ = 0.66	Sd = 0.73
			Min = 0	Max = 3
	12–24 months N = 319	57.1% (*n* = 182)	μ = 1.59	Sd = 1.73
			Min = 0	Max = 9
*Support for: R, Mo*	> 24 months N = 584	61.1% (*n* = 357)	μ = 2.15	Sd = 2.58
			Min = 0	Max = 14
Request for feedback (O)	≤ 12 months N = 119	10.9% (*n* = 13)	μ = 0.31	Sd = 1.07
			Min = 0	Max = 6
	12–24 months N = 319	23.5% (*n* = 75)	μ = 0.41	Sd = 0.92
			Min = 0	Max = 9
*Support for: R, F, Mo*	> 24 months N = 584	7.9% (*n* = 46)	μ = 0.16	Sd = 0.70
			Min = 0	Max = 8
**Form**	**Duration of e-portfolio use**	**In how many e-portfolios was the form present (%)**	**Descriptive Statistics:** How many learning goals were used per e-portfolio	
Learning goals and plans (Ma)	≤ 12 months N = 119	75.6% (*n* = 90)	μ = 5.32	Sd = 4.16
			Min = 0	Max = 17
	12–24 months N = 319	84.3% (*n* = 269)	μ = 11.47	Sd = 9.42
			Min = 0	Max = 49
*Support for: F, G, Mo*	> 24 months N = 584	93.2% (*n* = 544)	μ = 23.82	Sd = 17.12
			Min = 0	Max = 96

The percentage of e-portfolios that contained a particular form is also presented for each of the three cohorts. These figures show substantial percentages of non-use, even in the second- and third cohorts, indicating that there are GP trainees in their second- or third-year that miss a number of (mandatory) forms in their e-portfolio.

The table also shows if the pre-structured forms are mandatory or optional and if the form was, during design of the e-portfolio, envisioned to support the different SRL processes. No patterns in percentage of use or number of forms could be detected on basis of these characteristics.

### Phase two: content analysis

The results of the content analysis are displayed in Table 3. It shows that forms aimed at reflection were present in just over half of the e-portfolios (54.4%). In case an entry was present it could often (83.7%) not be qualified as reflective, because only experiences and events were described. Entries that were reflective did not exceed descriptive reflection (a singular explanation or justification was given for an event). In general, trainees described experiences with a positive focus, and did not discuss reasons, motives and context. For example: *“Things are going very well; well on track, tight schedule; meeting commitments”* and *“I could think more about the patient’s context, although sometimes I’m managing well enough already. I also know the families and their backgrounds and, if necessary, take this into consideration during the consult.”* There were no examples of dialogic or critical reflection in our sample: trainees did not document a variety of possible explanations for events, whereby also considering contextual influences.

**Table 3:** The percentage of e-portfolios that met the different SRL criteria for good practice from the codebook. The numbers in front of the criteria correspond to the item numbers of the codebook (Appendix A). The codebook describes the instructions used to decide if the criteria were met

Criteria from the codebook	How many e-portfolios fulfilled the criterium (%) N = 90
**Reflection**	
4. Presence of reflective forms	54.4%(*n* = 49)
5. If present, at what level	
Not reflective	83.7% (*n* = 41)
Descriptive reflection	16.3%(n = 8)
Dialogic reflection	0% (n = 0)
Critical reflection	0% (n = 0)
**Feedback Teacher**	
6. For which competences was feedback provided?	
Medical Expert	85.6%(*n* = 77)
Communicator	92.2%(*n* = 83)
Collaborator	87.7%(*n* = 79)
Leader	87.8%(n = 79)
Health Advocate	65.6%(*n* = 59)
Scholar	84.4%(*n* = 76)
Professional	94.4% (*n* = 85)
None	0% (n = 0)
7. Specificity *Was the feedback provided specific enough?*	27.8%(n = 25)
8. Focus *Did the feedback provided have an appropriate focus?*	91.1%(*n* = 82)
9. Purpose *Was the feedback provided in line with the purpose of the specific form?*	58.9%(n = 53)
10. Source *Were the criteria/source upon which the feedback was based clear?*	57.8%(*n* = 52)
11. Level *Did the provided feedback give insight into the level the trainee must attain?*	13.3% (*n* = 12)
**Feedback Supervisor**	
13. For which competences was feedback provided?	
Medical Expert	86.7%(*n* = 78)
Communicator	76.7%(*n* = 69)
Collaborator	37.8% (*n* = 34)
Leader	34.4%(n = 31)
Health Advocate	21.1%(*n* = 19)
Scholar	21.1%(n = 19)
Professional	36.7%(*n* = 33)
None	1.1%(n = 1)
14. Specificity *Was the feedback provided specific enough?*	35.6%(*n* = 32)
15. Focus *Did the feedback provided have an appropriate focus?*	87.8%(n = 79)
16. Purpose *Was the feedback provided in line with the purpose of the specific form?*	73.3%(*n* = 66)
17. Source *Were the criteria/source upon which the feedback was based clear?*	57.8%(n = 52)
18. Level *Did the feedback provided give insight in the level the trainee must attain?*	33.3%(n = 30)
**Goal-Setting and Planning**	
20. Specificity *Were the formulated learning goals specific?*	44.4%(*n* = 40)
21. Proximity *Were the formulated learning goals proximal (≤4 months)?*	23.3%(*n* = 21)
22. Congruence *Were the formulated learning goals in congruence with each other?*	87.8%(n = 79)
23. Challenging *Were the formulated learning goals challenging?*	97.8%(*n* = 88)
24. Origin *Were the formulated learning goals of a personal origin?*	64.4%(n = 58)
**Monitoring**	
25. Monitoring *Did the e-portfolio show signs of monitoring?*	74.4%(*n* = 67)

Feedback was in many cases confined to a summary of what went well or needed improvement, often lacking specificity, context and direction for future action. For example: *“Nice constructive attitude, friendly. Responds well to feedback.”* and “*Engages actively in the learning process, open for feedback. You are struggling with doubts about the discipline. Sometimes it is hard to keep one’s distance to the patient and not to take your work home.”* Feedback was specific in one quarter to one third of the e-portfolios (27.8 and 35.6% for teachers and supervisors, respectively). In about 90% of the e-portfolios, feedback was focused on performance and learning under the trainees’ control. Feedback was aligned with the purpose of the form in about two third of the e-portfolios (58.9% for teachers and 73.3% for supervisors). In these cases, the feedback concerned topics relevant for the competence at hand. A foundation or reasoning for the provided feedback was present in over half of the e-portfolios, such a ‘source’ for feedback was formulated in 57.8% of the e-portfolios. Tips for improvement were present in 13.3% (teachers) and 33.3% (supervisors) of the e-portfolios. Future-directed comments that were given generally missed specificity, for example: *“Now just demonstrate it”* and *“Keep taking (more) care of yourself here”.*

Learning goals and plans were specific in less than half (44,0%) of the e-portfolios, mainly because motives for the learning goals (‘why’) and a time or place indication (‘when’ and ‘where’) were missing. Trainees were able to formulate learning goals that are compatible with each other (87.8% of the e-portfolios) and they chose challenging goals to work with (97.8%). Almost two thirds of the e-portfolios contained learning goals with a personal origin. Notably, a number of learning goals appeared in numerous e-portfolios, for example, the placement of an intrauterine device and dealing with polypharmacy.

Signs of monitoring were present in three-quarters of the e-portfolios (74.4%). However, in one-third of these cases (24 of 67) the monitoring behaviour was limited, i.e. monitoring was mostly restricted to a feedback request, via a Mini-CEX, that related to one of the learning goals. In more elaborate cases of monitoring, trainees also noted the progress on learning goals, or adapted their original plans for goal attainment. In general, the themes and topics discussed in the feedback of teachers and reflections of trainees were unrelated to other components of the e-portfolio, such as the learning goals of trainees.

## Discussion

Portfolios are implemented to foster SRL of medical students and trainees, as SRL is considered an essential skill for those studying and/or working in the clinical setting [13, 14, 48]. It is expected that documenting reflections, learning goals and plans in a portfolio, stimulates the occurrence and depth of these SRL processes [2, 49, 50]. In addition, it is assumed that portfolio documentation, in which these reflections and learning goals are stored together with feedback from third parties, can help learners to self-assess their performance [51, 52] and with that offers the information needed to monitor one’s own learning process [12]. Common educational practice, such as the assessment of reflections and learning goals and the use of portfolio content as starting point for supervision (meetings), are based on these assumptions.

Our study shows, however, that the documentation of these SRL processes – reflection, feedback, goal-setting and planning, and monitoring – is limited within the e-portfolios of GP trainees. Previous studies have explained that (mandatory) written reflections have certain predicaments, that can potentially reduce reflection into a ‘tick-box-exercise’ which shows through documentation of shallow reflections [53–56]. An example of such a predicament is the apprehension that can be felt towards written reflections, i.e. the documentation of vulnerabilities, as such documentation might be considered harmful for assessment, professional development or legal issues [50, 56, 57]. Furthermore, our results concerning feedback are not uncommon, as the review of Bing-You et al. shows that more than a quarter of the 51 included articles on feedback exchange reported problems with low quality feedback, due to limited information, lack of specificity and absence of action plans [58]. With regard to goal-setting and planning previous studies have shown that learners hardly ever integrate goals into their workplace-based learning, unless they receive tailored coaching focused on effective goal-setting [59–62]. Although, the importance of monitoring of learning is often mentioned, there is still limited evidence of monitoring behaviour within medical education [45].

Considering the near absence of the included SRL processes in the e-portfolio content, could result in the simple explanation that trainees do not engage in SRL. However, a previous study using interviews, also targeting Dutch GP trainees, indicated that trainees did purposively regulate their learning [63]. The occurrence of SRL related cognitions, motivations and behaviour of trainees that probably took place - whether or not instigated by the e-portfolio - is apparently not captured in e-portfolio forms or content.

This could be explained by a variety of factors of some have been described above. In addition, trainees might not find the right words to accurately describe the SRL processes taking place. As SRL processes are complex, and might (partly) take place on a subconscious level [64], formulating if and how they were present asks for a high level of introspection and writing skills. Difficulties to adequately document the presence of SRL processes in a portfolio might be fuelled by another factor that is part of workplace-based medical education: a need to prioritise. When working in the clinical setting, where time pressure and a certain level of unpredictability are inevitable [14, 16, 65], trainees might not experience the time required to appropriately document the learning processes taking place. However, SRL processes may still occur in the head of trainees or be a topic of discussion with supervisors and teachers.

Another factor explaining the limited documentation of the different SRL processes might concern the difficulties that can occur when combining multiple purposes – assessment, accountability and support for SRL – in one portfolio [66]. The way the current GP e-portfolio was implemented might also have contributed. During implementation the focus was on assuring an appropriate functioning of the e-portfolio, especially regarding assessment, which needed to be covered sufficiently for accountability reasons. It was expected that the potential of the e-portfolio for the support and documentation of SRL would surface naturally when the e-portfolio was adequately used. Consequently, trainees received instructions concerning the use of the e-portfolio and its programmatic assessment function, but no specific instructions regarding the documentation of SRL.

With the choice to only provide information on the technical and assessment aspects of the e-portfolio we potentially overestimated the knowledge and skills that trainees and their teachers/supervisors have concerning (the documentation of) SRL. Research shows that support of a teacher/supervisor is important for the development of SRL [67], in addition, teachers/supervisors often do not know how to support SRL effectively [68, 69]. Thus, trainees, teachers and supervisors not only need to be supported in the technical aspects of a portfolio, but also need training concerning (documentation of) SRL, e.g. on effective reflection, feedback and goal-setting.

### Strengths and limitations

This is the first study to conduct content analysis on e-portfolios of medical trainees in order to achieve insights in the documentation of multiple SRL processes. For this purpose, a codebook, based on research literature, was constructed to rate the documentation of different criteria for good SRL practice in e-portfolio content.

To ensure anonymity of trainees, we chose to only include the pre-structured forms, which make up a considerable part of the e-portfolio. Consequently, it cannot be excluded that material stored in the folder for personal documents does show different patterns concerning SRL than those found in the pre-structured forms.

This study focused on self-assessment, via feedback and reflection, goal setting and planning, and monitoring, as these SRL processes are commonly mentioned in the health professions education literature in concern to portfolio use. However, SRL is a comprehensive concept that covers more than the processes that were considered within this research.

### Implications for future research

We focused on the documentation of SRL processes within e-portfolios, by using e-portfolio content as data source for quantitative analysis. Qualitative research would be valuable to assess how different factors, such as level of introspection and writing skills, influence the (limited) documentation of SRL processes. Likewise, other questions that still exist concerning the use of portfolios for the support of SRL require combined use of quantitative and qualitative measures. Future studies should, thus, use different data sources – e.g. portfolio content, portfolio users - and methods – e.g. questionnaires, observations, interviews - to attain further understanding of portfolio use for the support of SRL via triangulation. Thereby, clarifying to what extent and how portfolio use can support SRL.

## Conclusion

This study adds insights into the intricate relationship between portfolio use and SRL. Content analysis of e-portfolios used by GP trainees showed limited documentation of the SRL processes that are, according to health professions education literature, influenced by portfolio use: self-assessment, via reflection and feedback, goal-setting and planning, and monitoring. As the documentation of SRL in a portfolio asks for high level of introspection and writing skills, and requires time that is not always available within the clinical setting, one cannot expect documentation of SRL processes to appear in e-portfolio content without efforts.

## Supplementary information

**Additional file 1.** Appendix A. How is SRL documented in e-portfolio content. Codebook. In this appendix the codebook used for the content analysis is displayed.

**Additional file 2.** Appendix B. How is SRL documented in e-portfolio content. Interrater reliabilities. This table shows the interrater reliabilities that were calculated during the design of the codebook.

## Data Availability

The datasets used and/or analysed during the current study are available from the corresponding author on reasonable request.

## Abbreviations

SRL: Self-Regulated Learning
Electronic portfolio: e-portfolio
GP: General Practitioner
