# Supplementary material for: How is self-regulated learning documented in e-portfolios of trainees? A content analysis
Source: BMC Med Educ. 2020 Jun 26;20:205. doi: 10.1186/s12909-020-02114-4 (PMC7318487; doi:10.1186/s12909-020-02114-4)
Supplement: Supplementary file 1 — Additional file 1. Appendix A. How is SRL documented in e-portfolio content. Codebook. In this appendix the codebook used for the content analysis is displayed. [file 12909_2020_2114_MOESM1_ESM.docx]

**Appendix A**

Codebook e-portfolio analysis

**If the learning goals are added to the personal documents folder instead of a pre-structured form another portfolio is picked to rate.**

| **General**  1. This e-portfolio belongs to:  □ Female □ Male □ LUMC □ Radboudumc □ Maastricht University  □ 1^st^ year □ 2^nd^ year □ 3^rd^ year  2. Which year is rated?*  □ 1^st^ year □ 2^nd^ year □ 3^rd^ year  3. How many completed forms are available? .......  * For an e-portfolio that belongs to the 12-24 months cohort, the first year is coded.  For an e-portfolio that belongs to the 24-36 months cohort, the second year is coded.  For an e-portfolio that belongs to the >36 months cohorts, the third year is coded. |
| --- |

| **Reflection** (1, 2)  Preferably the most recent Compass, which is filled in by the trainee, is rated. The reflective questions at the end of the mini-CEX can be used when a Compass is missing.  4. Are forms containing reflections available? □ No *Skip question 5* □ Yes  5. What is the level of reflection?  □ No - Not reflective  *-Description of events that occurred/report of literature.*  *-No attempt to provide reasons/justification for events.*  □ Yes - Descriptive reflection  *- Reflective, not only a description of events but some attempt to provide reason or justification for events or actions, in a reportive or descriptive way. For example, " Chose this problem-solving activity because I believe that students should be active rather than passive learners."*  *- Two forms:*  *(a) Reflection based generally on one perspective/factor as rationale.*  *(b) Reflection is based on the recognition of multiple factors and perspectives* □ Yes - Dialogic reflection  *Demonstrates a ‘stepping back’ from the events/actions leading to a different level of mulling about, discourse with self and exploring the experience, events, and actions using qualities of judgements and possible alternatives for explaining and hypothesising. Such reflection is analytical or/and integrative of factors and perspectives and may recognise inconsistencies in attempting to provide rationales and critique.* □ Yes - Critical reflection  *Demonstrates an awareness that actions and events are not only located in, and explicable by, reference to multiple perspectives but are located in, and influenced by multiple historical, and socio-political contexts.* |
| --- |

| **Feedback Teachers** (3)  The most recent compass form, filled in by teachers, is rated.  6. For which of the competences is feedback provided? □ Medical expert *Medisch handelen* □ Health advocate *Maatschappelijk handelen* □ Communicator *Arts-patiënt communicatie*  □ Scholar *Kennis en wetenschap* □ Collaborator *Samenwerken*  □ Professional *Professionaliteit*  □ Leader *Organiseren*  □ None  7. Is the provided feedback *specific* enough? □ No *Two or more items (from the statement below) are structurally missing.* □ Yes *The ‘what’, ‘where’, ‘when’, ‘who’, ‘why’ and ‘how’ of the feedback is clear.*  8. Does the provided feedback have an appropriate *focus*? □ No *-Provided feedback is focused on trainees themselves and on their characteristics.   -Provided feedback is focused on general topics (such as medical information)*  □ Yes -*Provided feedback is focused on trainees’ performance and on their learning.   -Provided feedback is focused on actions under the trainees’ control*  9. Is the provided feedback in line with the *purpose* of the specific form? □ No *-Themes and topics relevant for the scope of the form and/or the competence at hand are discussed too minimally.*  *-Themes and topics outside the scope of the form and/or the competence at hand are discussed elaborately.* □ Yes *-Feedback is confined to the purpose of the form and/or the competence at hand.*  10. Are the criteria/*source* upon which the feedback was based clear? □ No *There is no reasoning and/or funding for the provided feedback.* □ Yes *There are things mentioned regarding how the feedback came about.*  11. Does the provided feedback give insight in the *level* the trainee must attain?  □ No *The feedback only mentions topics that went right/wrong and/or are in need of extra attention.*  □ Yes *The feedback (also) mentions what the trainee can do to improve current behaviour.* |
| --- |

| **Feedback Supervisor** (3)  For supervisors preferably the most recent mini-CEX is rated, if this form is not available a Compass-form can be looked into as well.  NB: this question was eliminated for definitive rating due to insufficient IRR  12. For which of the competences is feedback requested?  □ Medical expert *Medisch handelen* □ Health advocate *Maatschappelijk handelen* □ Communicator *Arts-patiënt communicatie*  □ Scholar *Kennis en wetenschap* □ Collaborator *Samenwerken*  □ Professional *Professionaliteit*  □ Leader *Organiseren*  □ None  13. For which of the competences is feedback provided? □ Medical expert *Medisch handelen* □ Health advocate *Maatschappelijk handelen* □ Communicator *Arts-patiënt communicatie*  □ Scholar *Kennis en wetenschap* □ Collaborator *Samenwerken*  □ Professional *Professionaliteit*  □ Leader *Organiseren*  □ None  14. Is the provided feedback *specific* enough? □ No *Two or more items (from the statement below) are structurally missing.* □ Yes *The ‘what’, ‘where’, ‘when’, ‘who’, ‘why’ and ‘how’ of the feedback is clear.*  15. The provided feedback has an appropriate *focus*? □ No *-Provided feedback is focused on trainees themselves and on their characteristics.   -Provided feedback is focused on general topics (such as medical information).*  □ Yes *-Provided feedback is focused on trainees’ performance and on their learning.   -Provided feedback is focused on actions under the trainees’ control.*  16. Is the provided feedback in line with the *purpose* of the specific form? □ No *-The question asked by the trainee is not answered in the feedback.(Requested feedback).   -Themes and topics unrelated to the question of the trainee are discussed elaborately.(Requested feedback).   -Themes and topics relevant for the scope of the form and/or the competence at hand are   discussed too minimally. (Periodical feedback).   -Themes and topics outside the scope of the form and/or the competence at hand are discussed elaborately. (Periodical feedback).*   □ Yes *-The question of the trainee is answered appropriately (Requested feedback).   -Feedback is confined to the purpose of the form and/or the competence at hand. (Periodical*  *feedback).*  17. Are the criteria/*source* upon which the feedback was based clear? □ No *There is no reasoning and/or funding for the provided feedback.* □ Yes *There are things mentioned regarding how the feedback came about.*  18. Does the provided feedback give insight in the *level* the trainee must attain?  □ No *The feedback only mentions topics that went right/wrong and/or are in need of extra attention.*  □ Yes *The feedback (also) mentions what the trainee can do to improve current behaviour.* |
| --- |

| **Goal-setting and planning** (4)  19. How many learning goals are available?  ...... learning goals on ...... forms  The most recent form containing learning goals is rated.  20. Are the formulated learning goals *specific*? □ No *Two or more items (from the statement below) are structurally missing.*  □ Yes *The ‘what’, ‘where’, ‘when’, ‘who’, ‘why’ and ‘how’ of the learning goals is clear.*  21. Are the formulated learning goals *proximal*?  □ No -*Time frame for the learning goals is >4 months (the interval between two progress meetings).   -The learning goals have no time frame at all.*   □ Yes *The learning goals are set for the coming 4 months.*   22. Are the formulated learning goals in *congruence* with each other?  □ No *-The learning goals are not compatible with each other.   -The learning goals are not attainable, because they are as a whole too time-consuming.* □ Yes *It is attainable to achieve the learning goals within the set period.*  23. Are the formulated learning goals *challenging*? □ No *The goal can be achieved without any additional effort.*   □ Yes *-Personal effort is needed in order to achieve the goal.*   *-The formulated learning goals cover a multitude of competencies and fields.*  24. Are the formulated learning goals of a personal *origin*? □ No *The learning goals coincide with curricular objectives.*  □ Yes -*The learning goals are different from curricular objectives.  -The learning goals coincide with curricular objectives, but the trainee elucidates why special attention is needed for this topic.* |
| --- |

| **Monitoring**  25. Does the e-portfolio show signs of *monitoring*?  □ No  □ Yes *themes/topics recur throughout different forms, learning goals mention motives derived from practice and/or are followed up on, ....*  Make note of the observed mechanisms of monitoring  ................................................................................................................................................................................................................................................................................................................................................................................................................................................................................................................................................................................................................ |
| --- |

| **Memos**  ............................................................................................................................................................................................................................................................................................................................................................................................................................................................................................................................................................................................................................................................................................................................................................................................................................................................................................................................................................................................................................................................................................................................................................................................................................................................................................................................................................................................................................................................................................................................................................................................................................................................................................................................................................................................................................................................................................................................................................................................................................................................................................................................................................................................................................................................................................................................................................................................................................................................................................................................................................................................................................................................................................................................................................................................................................................................................................................................................................................................................................................................................................................................................................................................................................................................................................................................................................................................................................................................................................................................................ |
| --- |

**References**

1. Hatton N, Smith D. Reflection in teacher education: Towards definition and implementation. Teaching and teacher education. 1995;11(1):33-49.

2. Pee B, Woodman T, Fry H, Davenport ES. Appraising and assessing reflection in students' writing on a structured worksheet. Med Educ. 2002;36(6):575-85.

3. Gibbs G, Simpson C. Conditions under which assessment supports students’ learning. Learning and teaching in higher education. 2005(1):3-31.

4. Zimmerman BJ. Goal setting: A key proactive source of academic self-regulation. Motivation and self¬ regulated learning: Theory, research, and applications. 2008;267.
