# Supplementary material for: How is self-regulated learning documented in e-portfolios of trainees? A content analysis
Source: BMC Med Educ. 2020 Jun 26;20:205. doi: 10.1186/s12909-020-02114-4 (PMC7318487; doi:10.1186/s12909-020-02114-4)
Supplement: Supplementary file 2 — Additional file 2. Appendix B. How is SRL documented in e-portfolio content. Interrater reliabilities. This table shows the interrater reliabilities that were calculated during the design of the codebook. [file 12909_2020_2114_MOESM2_ESM.docx]

**Appendix B**

| Item | IRR measurement 1 n=24 | IRR measurement 2 n=10 | IRR measurement3 n=20 |
| --- | --- | --- | --- |
| General | | | |
| 1. Sex | 1.0 – 100% | 1.0 – 100% | 1.0 – 100% |
| 1. Institute | 1.0 – 100% | 1.0 – 100% | 1.0 – 100% |
| 1. Year of training | 1.0 – 100% | 1.0 – 100% | 0.94 – 95% |
| 2. Year of rating | 1.0 – 100% | 1.0 – 100% | 1.0 – 100% |
| 3. Completed forms | 0.98 – 50% | 1.0 – 100% | 0.99 – 90% |
| Reflection | | | |
| 4. Presence | 0.41 – 54.2% | 1.0 – 100% | 0.67 – 85% |
| 5. Level | 0.76 – 83.3% | 1.0 – 100% | 0.84 – 80% |
| Feedback Teachers | | | |
| 6. Medical Expert | 0.85 – 70.8% | 0.75 – 90% | 1.0 – 100% |
| 6. Communicator | 1.0 – 100% | 1.0 – 100% | 1.0 – 100% |
| 6. Collaborator | 1.0 – 100% | 1.0 – 100% | 1.0 – 100% |
| 6. Leader | 0.65 – 75% | 1.0 – 100% | 1.0 – 100% |
| 6. Health Advocate | 0.88 – 75% | 1.0 – 100% | 1.0 – 100% |
| 6. Scholar | 0.85 – 75% | 1.0 – 100% | 1.0 – 100% |
| 6. Professional | 1.0 – 100% | 0.63 – 90% | 1.0 – 100% |
| 6. None | 1.0 – 100% | 1.0 – 100% | 1.0 – 100% |
| 7. Specificity | 0.73 – 70.8% | 1.0 – 100% | 1.0 – 100% |
| 8. Focus | 0.48 – 66.7% | 1.0 – 100% | 0.78 – 85% |
| 9. Purpose | 0.66 – 66.7% | 0.42 – 70% | 0.70 – 85% |
| 10. Source | 0.29 – 54.2% | 1.0 – 100% | 0.70 – 85% |
| 11. Level | 0.61 – 70.8% | 1.0 – 100% | 0.0 – 100% |
| Feedback Supervisor | | | |
| 12. Medical Expert | 0.45 – 54.2% | Eliminated | Eliminated |
| 12. Communicator | 0.13 – 33.3% | Eliminated | Eliminated |
| 12. Collaborator | 0.44 – 50% | Eliminated | Eliminated |
| 12. Leader | 0.18 – 50% | Eliminated | Eliminated |
| 12. Health Advocate | 0.13 – 50% | Eliminated | Eliminated |
| 12. Scholar | 0.18 – 50% | Eliminated | Eliminated |
| 12. Professional | 0.11 – 37.5% | Eliminated | Eliminated |
| 12. None | 0.64 – 62.5% | Eliminated | Eliminated |
| 13. Medical Expert | 0.61 – 70.8% | .63 – 90% | 1.0 – 100% |
| 13. Communicator | 0.77 – 70.8% | 1.0 – 100% | 1.0 – 100% |
| 13. Collaborator | 0.76 – 70.8% | 0.75 – 90% | 0.90 – 95% |
| 13. Leader | 1.0 – 100% | 0.79 – 90% | 1.0 – 100% |
| 13. Health Advocate | 1.0 – 100% | 1.0 – 100% | 1.0 – 100% |
| 13. Scholar | 0.77 – 75% | 0.63 – 100% | 1.0 – 100% |
| 13. Professional | 0.56 – 66.6% | 0.79 – 90% | 1.0 – 100% |
| 13. None | 0.00 – 75% | 1.0 – 100% | 1.0 – 100% |
| 14. Specificity | 0.56 – 66.7% | 1.0 – 100% | 0.90 – 95% |
| 15. Focus | 0.77 -75% | 1.0 – 100% | 0.62 – 90% |
| 16. Purpose | 0.76 – 70.8% | 0.62 – 80% | 0.83 – 95% |
| 17. Source | 0.03 – 41.7% | 1.0 – 100% | 0.88 – 95% |
| 18. Level | 0.54 – 62.5% | 1.0 – 100% | 0.90 – 95% |
| Goal-Setting and Planning | | | |
| 19. Number of goals | 0.85 – 91.7% | 1.0 – 100% | 1.0 – 100% |
| 19. Number of forms | 0.86 – 95.8% | 0.96 – 90% | 1.0 – 100% |
| 20. Specificity | 0.43 – 79.2% | 1.0 – 100% | 0.57 – 80% |
| 21. Proximity | 0.69 – 91.7% | 0.81 – 90% | 0.46 – 90% |
| 22. Congruence | 0.65 – 95.8% | 1.0 – 100% | 0.62 – 95% |
| 23. Challenging | 0.48 – 87.5% | 0.63 – 90% | 0.49 – 85% |
| 24. Origin | 0.25 – 75% | 0.75 – 90% | 0.67 – 85% |
| Monitoring | | | |
| 25. Monitoring | n/a | 0.63 – 90% | 0.62 – 90% |

Interrater reliabilities (IRR) of the different items of the codebook using Krippendorff’s alpha and percentage of agreement. Black results refer to satisfactory IRR (>0.67), orange results are sufficient (≥0.60 - ≤0.67) and red results are insufficient (<0.60).
